# Supplementary material for: Vector-borne Trypanosoma brucei parasites develop in artificial human skin and persist as skin tissue forms
Source: Nat Commun. 2023 Nov 23;14:7660. doi: 10.1038/s41467-023-43437-2 (PMC10667367; doi:10.1038/s41467-023-43437-2)
Supplement: Supplementary file 1 — Supplementary Information [file 41467_2023_43437_MOESM1_ESM.pdf]

Supplementary Information for

Vector-borne *Trypanosoma brucei* parasites develop in artificial human skin and persist as skin tissue forms

Christian Reuter, Laura Hauf, Fabian Imdahl, Rituparno Sen, Ehsan Vafadarnejad, Philipp Fey, Tamara Finger, Nicola G. Jones, Heike Walles, Lars Barquist, Antoine-Emmanuel Saliba, Florian Groeber-Becker, Markus Engstler

Correspondence to: markus.engstler@biozentrum.uni-wuerzburg.de

This PDF file includes:

Supplementary Note
Supplementary Figs. 1-9
Supplementary References

Supplementary Note:

Improved mechanical properties reduce dermal contraction and weight loss in primary human skin equivalents

A major problem with collagen-based skin equivalents is their susceptibility to fibroblast-mediated contraction during culture. The poor mechanical stability of the dermal component[1] makes standardization difficult. In this study, we refined a skin equivalent[2] by improving the mechanical stability of the dermal component to reach a high level of standardization and reproducibility. A computer-assisted compression system consisting of a custom-made compression reactor (Supplementary Fig. 1a, b) and a linear motor (Supplementary Fig. 1c) was used to compress the dermal component by a factor of 7. The high-density dermal equivalents (hdDEs) were characterized by a diameter of 12 mm, a height of 1 mm, and a calculated collagen concentration of 46.9 mg/ml. The increased collagen concentration and reduced water content improved the mechanical properties of the hdDEs, as shown by rheology. Strain sweep measurements confirmed a 3-fold higher storage modulus (G′) in the linear viscoelastic region of cell-free hdDEs compared to cell-free, non-compressed low-density dermal equivalents (ldDEs; ccollagen = 6.7 mg/ml), indicating stronger viscoelastic strength of the cell-free hdDEs (Fig. 1b and Supplementary Fig. 1d). The viscoelastic strength was even more pronounced in hdDEs colonized with normal human dermal fibroblasts (NHDF) for 23 days. Furthermore, ldDEs showed an earlier flow point (G′ = G″) than hdDEs, correlating with a lower structural resistance against shear forces (Supplementary Fig. 1d). Compared to human skin biopsies, G′ was 11-fold lower in cell-free ldDEs, while only 3-fold lower in hdDEs (Fig. 1b and Supplementary Fig. 1d). In contrast to hdDEs, skin biopsies additionally included the epidermal skin layer, indicating an even smaller difference in mechanical stability between pure human dermis and hdDEs.
Long-term comparison of hdDEs and ldDEs confirmed that hdDEs show significantly less contraction and weight loss over time, while having a similar number of proliferative NHDF (Supplementary Fig. 1e, and Supplementary Fig. 2a, b). While hdDEs lost only 6.7 % of weight and 15.7 % of area during 3 weeks of culture, ldDEs lost more than 82.7 % and 71.8 %, respectively. Scanning electron microscopy confirmed proper assembly of collagen fibers in both DEs (Supplementary Fig. 2c). However, in contrast to ldDEs, the collagen fibers in hdDEs showed a denser and more uniform distribution.

High-density skin equivalents recapitulate key anatomical, cellular, and functional aspects of native human skin

High-density dermal equivalents were colonized with normal human epidermal keratinocytes (NHEK) on day 3 and serial histological analysis showed that the morphology of high-density skin equivalents (hdSEs) on day 15 resembled that of native human skin, with a fully differentiated epidermis in which the 4 characteristic layers had formed (Fig. 1c, a-d). The epidermis was stable until day 23, and a prolonged culture was possible but ultimately led to a *stratum corneum* with aspects of hyperkeratosis. Thus, in a time window of minimally 9 days, the tissue architecture of the hdSE resembled native human skin.
In addition, a comparative analysis showed that physiological skin markers were expressed in the hdSE at day 23 (Supplementary Fig. 2d). The presence of epidermal differentiation markers such as KRT5 and KRT14 in the basal layer, KRT10 in the suprabasal layers, and loricrin, filaggrin, and involucrin in the *stratum granulosum* and *stratum corneum* verified an anatomically correct

differentiation of the epidermis. The presence of collagen IV demonstrated the presence of basement membrane proteins at the structurally and biochemically complex dermal-epidermal junction. Moreover, the homogeneous distribution of vimentin-positive cells in the dermis indicated the presence of NHDF.

To define the cellular heterogeneity of the hdSE in more detail scRNAseq was performed using the droplet-based 10x Genomics platform[3] (Supplementary Fig. 3a). Data analysis of 5958 cells that passed quality control (Supplementary Fig. 3b and Supplementary Data 1) resulted in a t-distributed stochastic neighbor embedding (t-SNE) plot displaying 7 clusters with distinct expression profiles (Fig. 1d and Supplementary Data 1).

All clusters were confirmed by the expression of known marker genes (Fig. 1e). Fibroblasts were identified in two clusters (clusters FB and FB-Pro) by their archetypal markers DCN and LUM[4]. SFRP2 and DPP4 expression was detected in the FB cluster, characterizing the most abundant major fibroblast population within the dermis. Cells expressing these markers are known to be small and elongated and express high levels of extracellular matrix components[5]. Keratinocytes were detected in 5 clusters and their diversity was mainly due to their degree of differentiation. Basal keratinocytes (clusters B-I, B-II, B-III, and B-Pro) highly expressed KRT5 and KRT14, whereas suprabasal keratinocytes (S) specifically expressed KRT1 and KRT10. Proliferating cells were present in both cell populations. They were identified by their expression of mitotic markers such as MKI67 and TOP2A (clusters FB-Pro and B-Pro).

Differential gene expression analysis of the 5 keratinocyte subclusters revealed 3 clusters of basal keratinocytes (B-I, B-II, and B-III), which is in good agreement with native human skin[6]. The B-I cluster was, besides KRT5 and KRT14, also characterized by KRT15, DAPL1, and CXCL14. CXCL14 is constitutively expressed in skin and produced by keratinocytes[7]. Cluster B-II contained mainly genes that are important for the formation of hemidesmosomes and the attachment of the epidermis to the dermis via the basement membrane such as LAMA3, LAMB3, LAMC2, ITGA6, ITGB4, DST, PLEC, COL7A1, and COL17A1 (Fig. 1f, Supplementary Fig. 3c, and Supplementary Data 1).

Cluster B-III was defined by RHCG, KRT6A, and KRT16, similar to what was found earlier, and it was assumed that these cells could directly differentiate into suprabasal cells[6]. Suprabasal cells in cluster S expressed known markers for epidermal differentiation such as SBSN or KRTDAP. Cluster B-Pro was enriched for the cell cycle markers UBE2C and PCNA. Furthermore, a detailed analysis of extracellular matrix (ECM)-associated genes revealed a remarkable repertoire of expressed ECM genes in the hdSE (Supplementary Fig. 3c). Many components of the ECM, including the collagens COL1A1, COL1A2, COL3A1, COL12A1, as well as elastin (ELN), fibronectin (FN1), and fibrillin (FBN1) were expressed in the fibroblast cluster. The same was true for factors involved in matrix assembly, such as MFAP4, SFRP2, LOX, FAP, and ANXA2, as well as for proteins involved in matrix remodeling, such as matrix metallopeptidases (MMP2, MMP14) and TIMP metallopeptidase inhibitors (TIMP1, TIMP3).

Different culture media were tested to find optimal conditions for co-cultivation of parasites and skin equivalents (Supplementary Fig. 4a, b). Moreover, to test whether primary cells isolated from different human donors are compatible with each other, hdSEs consisting of NHDF and NHEK from three different donors were evaluated based on the thickness of the cellular epidermis (Supplementary Fig. 4b, c). Overall, these results indicate that trypanosomes and hdSEs can be co-cultured with a mixture of trypanosome and skin medium without detrimental effects.

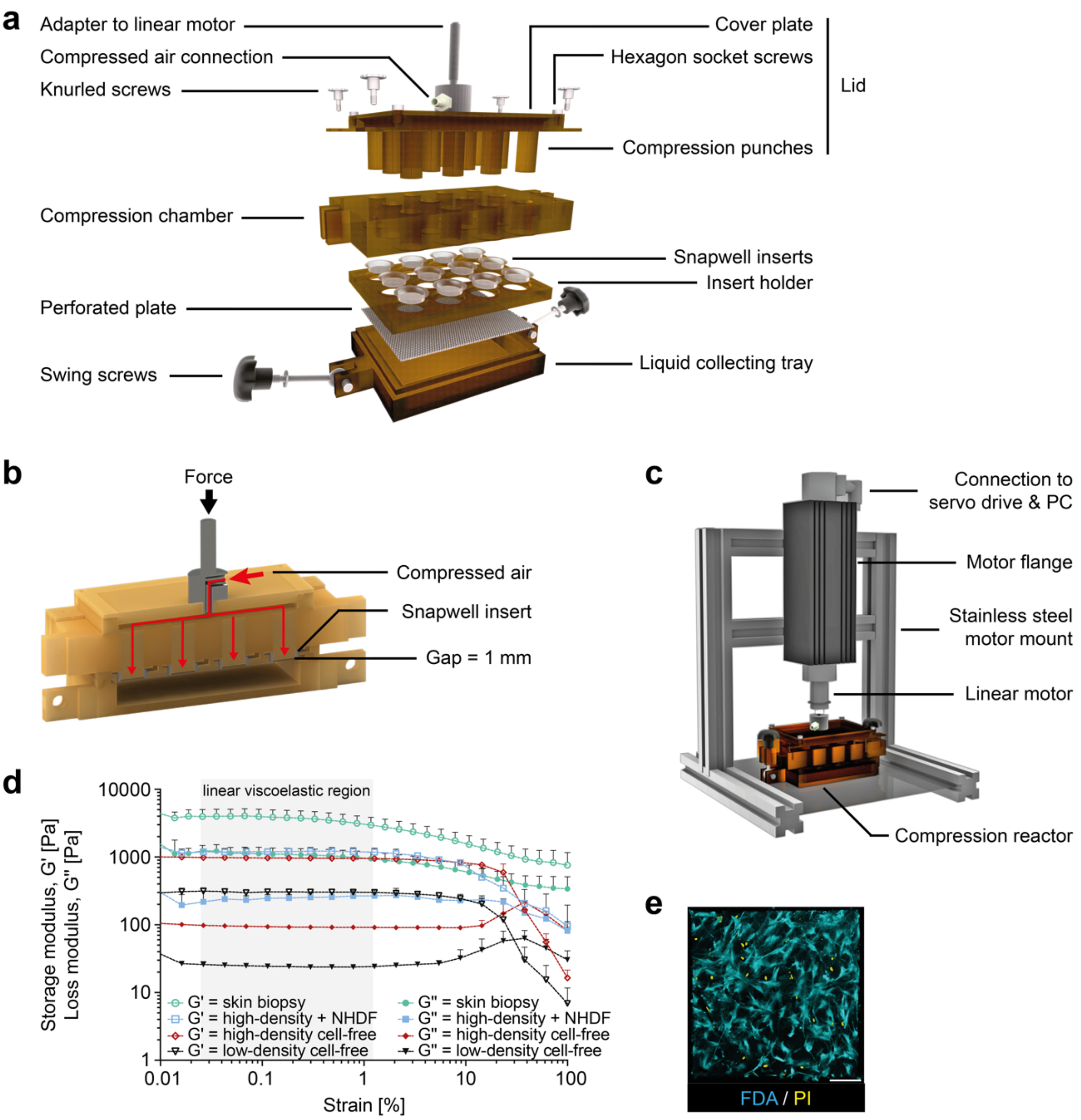

Supplementary Figure 1. Bioreactor design and compression system. (a) Representation of the computer-aided design (CAD) of the compression reactor with all individual parts. **(b)** Cross section through the closed reactor after complete compression. The red arrows mark the route of the compressed air through the reactor. The compressed air was introduced through the compressed air connection, which could then exit through the channels in the individual compression punches to release the dermal equivalents. **(c)** Representation of the compression system. Shown is the configuration of the linear motor, which was attached to a stainless-steel motor mount via the motor flange. The motor can be controlled with the LinMot®-Talk software by connecting it to a computer. **(d)** Plot of oscillatory rheological measurements. Storage, G′ (open shapes), and loss modulus, G″ (filled shapes), of cell-free and normal human dermal fibroblast (NHDF)-populated high- and low-density dermal equivalents as well as skin biopsies were determined in dependency on the strain deformation (ω = 6.28 rad/s, T = 22 °C). The linear viscoelastic region (gray box) was determined

according to DIN 53019-4. Data represent means ± SD (low-density cell-free, n = 3; high-density cell-free, n = 3; high-density + NHDF, n = 6; skin biopsies, n = 7). Source data are provided as a Source Data file. **(e)** Assessment of NHDF viability in high-density dermal equivalents on day 23 after compression. NHDF were stained with fluorescein diacetate (FDA, cyan) and propidium iodide (PI, yellow) for live/dead discrimination. Scale bar, 100 µm.

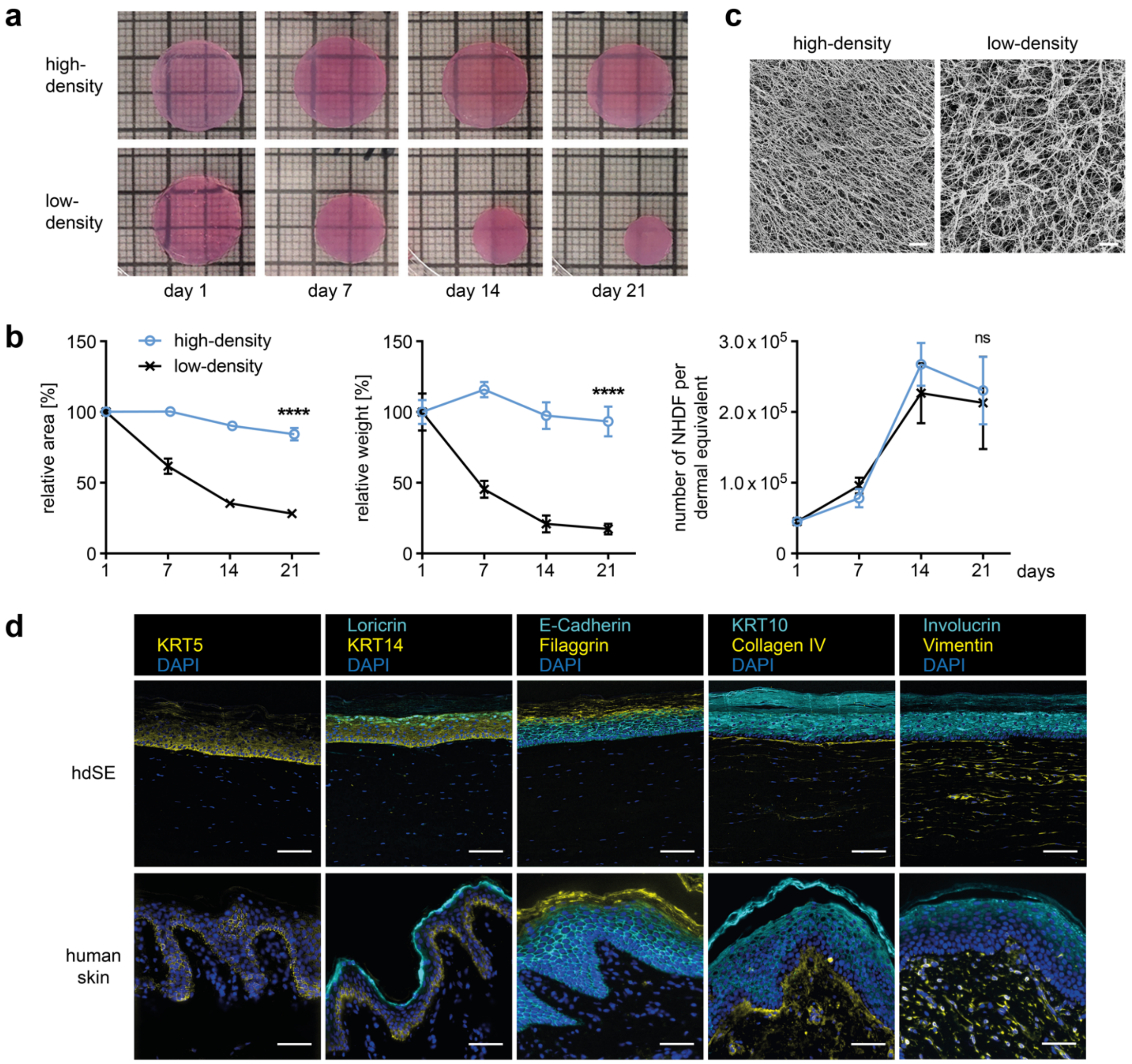

Supplementary Figure 2. High-density dermal equivalents have improved mechanical properties. (a) Images of normal human dermal fibroblast (NHDF)-populated high-density dermal equivalents (hdDEs) and low-density dermal equivalents (ldDEs) at various culture times show massive shrinkage of ldDEs (day 21: Ø = 12 mm versus 7 mm). **(b)** Quantification of the relative area and weight, and the number of NHDF per dermal equivalent of hdDEs and ldDEs. Graphs represent means ± SD (n = 9 – 24 individual models). Unpaired t-test, **** $p < 0.0001$, ns, not significant. Source data are provided as a Source Data file. **(c)** Scanning electron microscopy of the fibrillar collagen ultrastructure of hdDEs and ldDEs show a denser collagen network in hdDEs. Scale bar, 2 µm. **(d)** Distinctive physiological skin markers were detectable in high-density skin equivalents (hdSEs). At day 23, tissues were stained with antibodies against loricrin, E-cadherin, Cytokeratin 10 (KRT10), involucrin (all cyan), and Cytokeratin 5 (KRT5), Cytokeratin 14 (KRT14), filaggrin, collagen IV, and vimentin (all yellow). DNA was visualized with DAPI (blue). Images were acquired by fluorescence microscopy of hdSE cross sections. Scale bar, 75 µm.

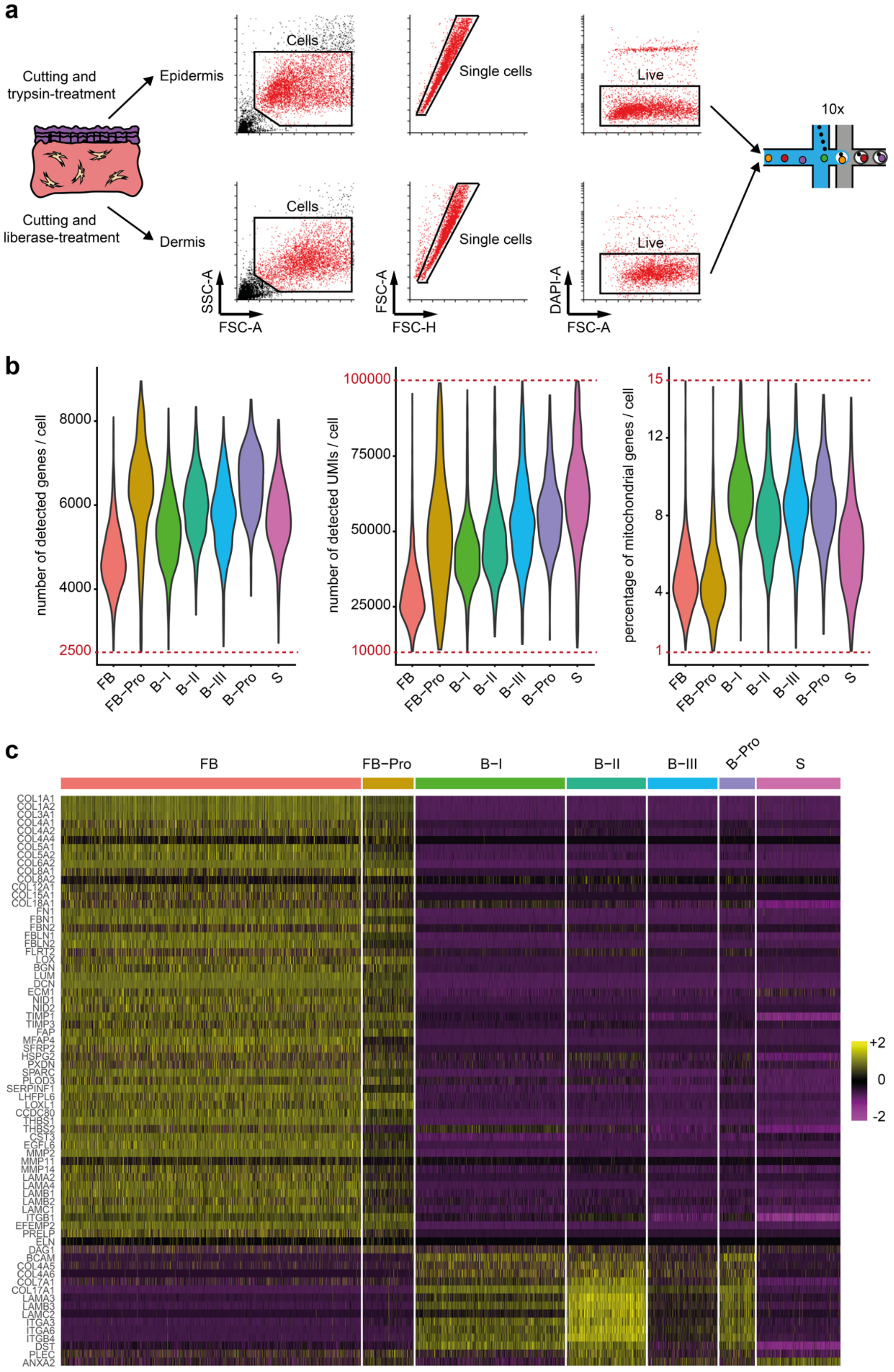
a
Cutting and trypsin-treatment
Epidermis
Cutting and liberase-treatment
Dermis
Cells
Single cells
Live
SSC-A
FSC-A
FSC-H
DAPI-A
10x
b
number of detected genes / cell
number of detected UMIs / cell
percentage of mitochondrial genes / cell
FB
FB−Pro
B−I
B−II
B−III
B−Pro
S
c
FB
FB−Pro
B−I
B−II
B−III
B−Pro
S
COL1A1
COL1A2
COL3A1
COL4A1
COL4A2
COL4A4
COL5A1
COL5A2
COL6A2
COL8A1
COL8A2
COL12A1
COL15A1
COL18A1
FN1
FBN1
FBN2
FBLN1
FBLN2
FLRT2
LOX
BGN
LUM
DCN
ECM1
NID1
NID2
TIMP1
TIMP3
FAP
MFAP4
SFRP2
HSPG2
PXDN
SPARC
PLOD3
SERPINF1
LHFPL6
LOXL1
CCDC80
THBS1
THBS2
CST3
EGFL6
MMP2
MMP11
MMP14
LAMA2
LAMA4
LAMB1
LAMB2
LAMC1
ITGB1
EFEMP2
PRELP
ELN
DAG1
BCAM
COL4A5
COL4A6
COL7A1
COL17A1
LAMA3
LAMB3
LAMC2
ITGA3
ITGA6
ITGB4
DST
PLEC
ANXA2
+2
0
-2

Supplementary Figure 3. Strategy and quality control of the single-cell RNA sequencing experiment of high-density skin equivalents and analysis of the expression of extracellular matrix-associated genes. (a) The epidermis and dermis of high-density skin equivalents were separated, cut, and dissociated by trypsin or liberase treatment. Cell suspensions were separated by fluorescence-activated cell sorting and single, live cells were sorted. Subsequently, cells were loaded on the droplet-based 10x platform according to the manufacturers' instructions and sequenced. **(b)** Quality control of 10x data showing the number of detected genes, UMIs (unique molecular identifiers), and the percentage of mitochondrial (mt) genes per cell in each of the 7 subclusters (fibroblasts (FB), proliferating fibroblasts (FB-Pro), basal (B-I - III), suprabasal (S), and proliferating keratinocytes (B-Pro)). The red lines in each graph represent quality control thresholds and cells above or below these thresholds were excluded from analysis. **(c)** Heatmap showing the scaled expression levels of extracellular matrix-associated genes differentially expressed in each cluster. The color key from pink to yellow indicates low to high gene expression levels. Each column represents a single cell, each row represents an individual gene. Gene names are listed to the left.

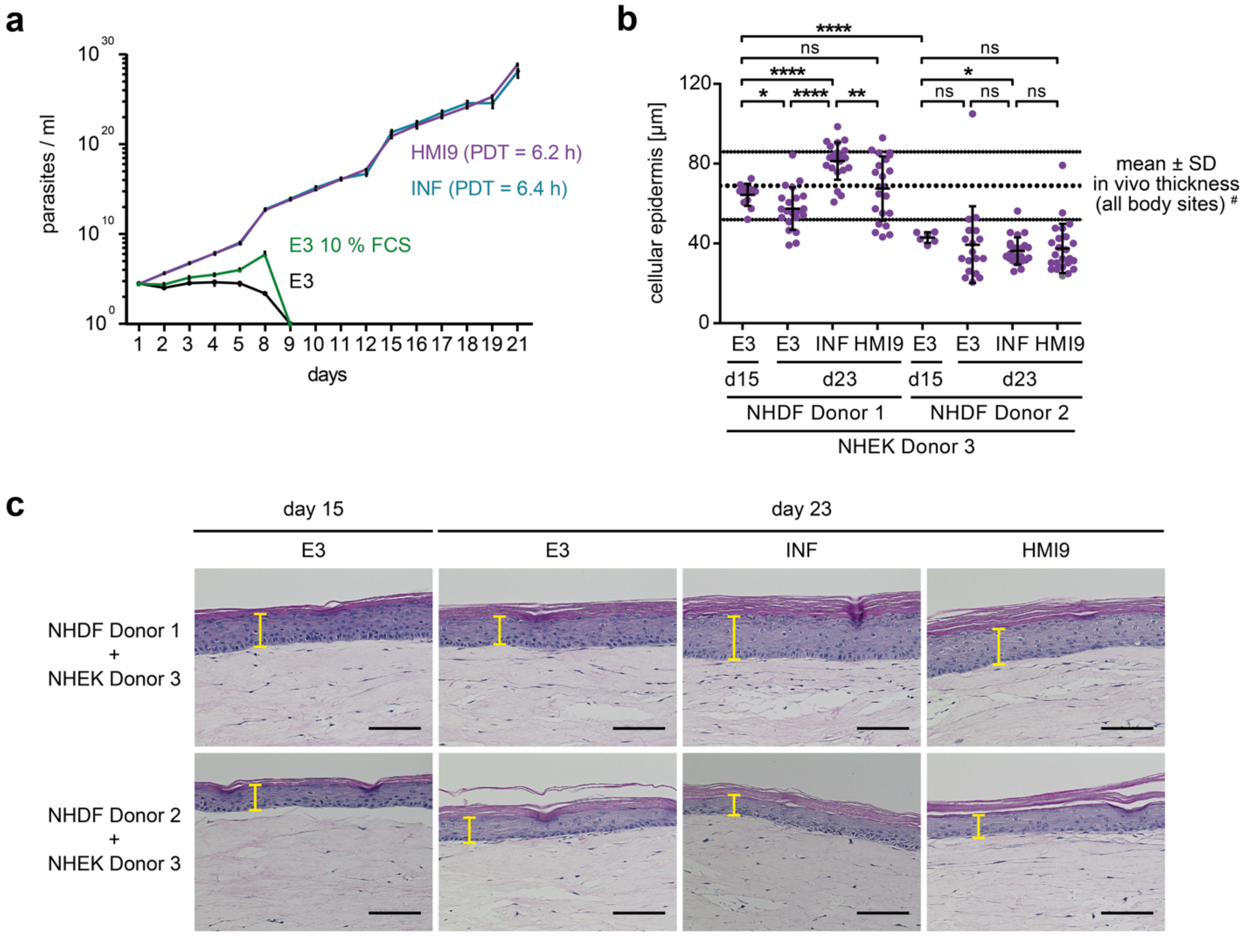

Supplementary Figure 4. ***T. brucei*** **parasites and high-density skin equivalents can be cultured with the same medium without growth deficits. (a)** Growth and population doubling times of monomorphic Lister 427 MITat 1.2 13-90 strain parasites in various media: trypanosome medium HMI9 (purple), skin medium E3 supplemented with 10 % FCS (green) or without FCS (black), and a mixture of both media (= INF, cyan). Data represent means ± SD (n = 3 individual cultures). Source data are provided as a Source Data file. **(b)** Analysis of the thickness of the cellular epidermis of high-density skin equivalents (hdSEs) generated with normal human dermal fibroblasts (NHDF) from two different donors (donor 1 and 2) and normal human epidermal keratinocytes (NHEK) from a third donor (donor 3). Skin equivalents were cultured with E3 medium until day 15. On day 15, the medium was changed to E3, INF, or HMI9 until day 23. ImageJ was used to measure the thickness of the cellular epidermis in hematoxylin and eosin-stained cross sections of hdSEs. Data represent means ± SD. Unpaired t-test, **** $p < 0.0001$, ** $p < 0.01$, * $p < 0.05$, ns, not significant. Source data are provided as a Source Data file. **(c)** Hematoxylin and eosin-stained cross sections of hdSEs cultured with E3, INF, or HMI9 media at day 23 compared with day 15. The yellow markings indicate the thickness of the cellular epidermis. Scale bar, 100 µm.

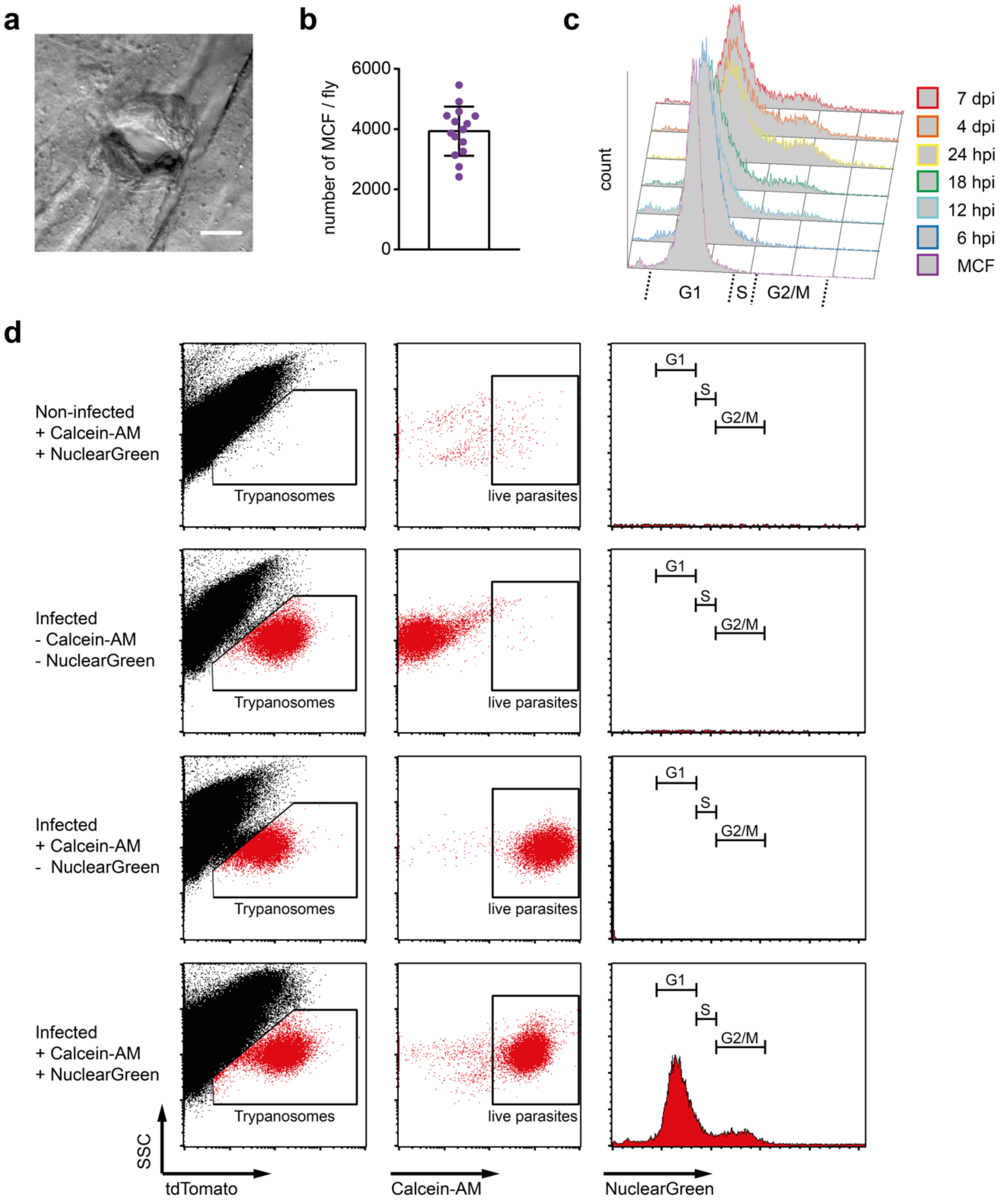

Supplementary Figure 5. Epidermal wound, number of tsetse-transmitted parasites and fluorescence-activated cell sorting gating strategy. (a) Stereo microscopy of the epidermal wound caused by the fly bite. Scale bar, 50 µm. **(b)** Number of metacyclic forms (MCF) in spit samples from individual salivary gland-positive flies. The maximum number of injectable MCF per fly bite was 3934 on average. Results are shown as mean ± SD (n = 15 individual flies). Source data are provided as a Source Data file. **(c)** Representation of cell cycle histograms of the flow cytometric

assessment of the cell cycle distribution of skin-residing trypanosomes at various timepoints post-infection. **(d)** Flow cytometric profiles showing the gating strategy to detect trypanosomes (tdTomato positive) isolated from skin equivalents, to determine parasite viability (Calcein-AM positive), and to assess cell cycle distribution with NuclearGreen staining.

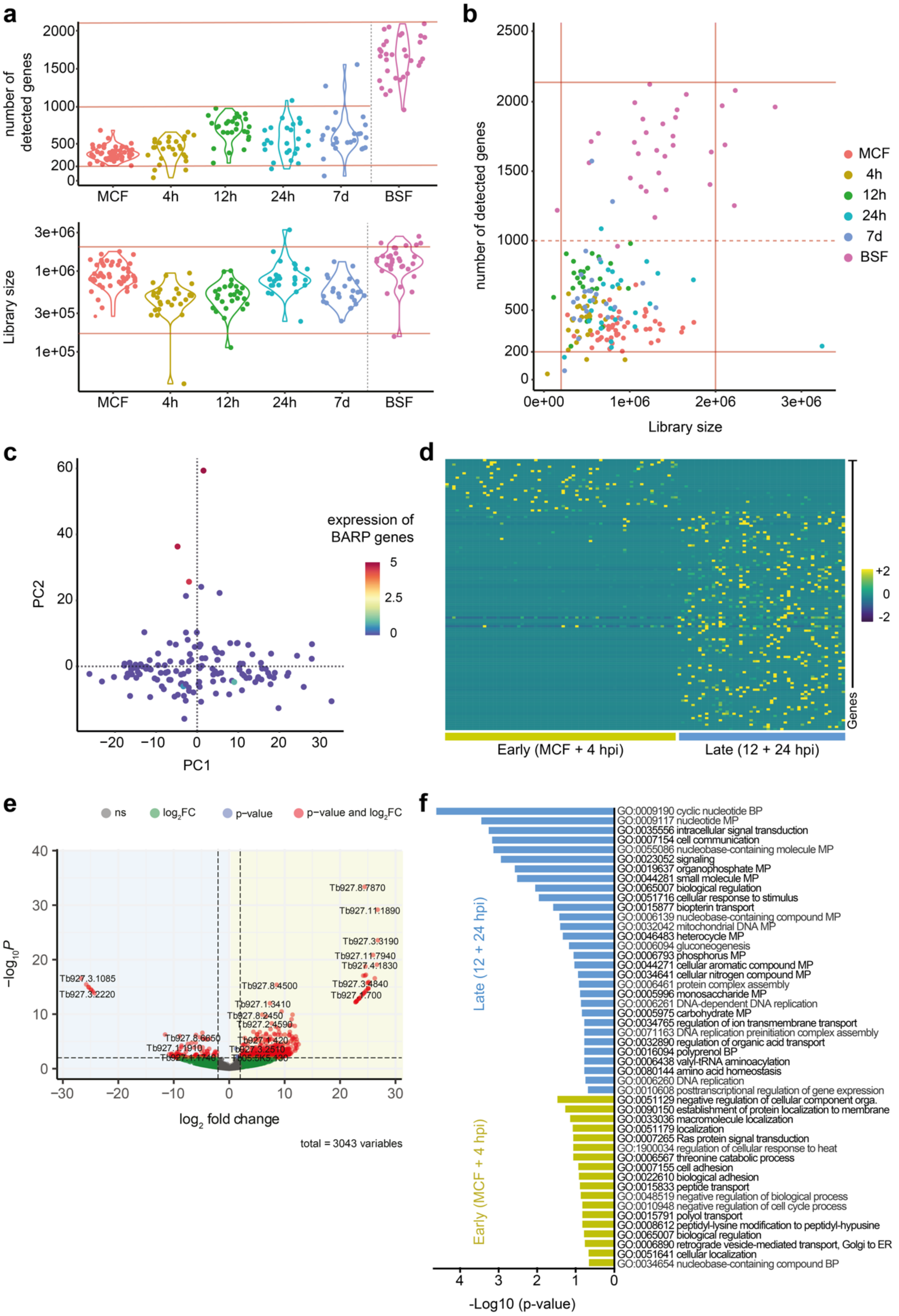
a
number of detected genes
2000
1500
1000
500
200
0
MCF
4h
12h
24h
7d
BSF
Library size
3e+06
1e+06
3e+05
1e+05
MCF
4h
12h
24h
7d
BSF
b
number of detected genes
2500
2000
1500
1000
500
200
0
0e+00
1e+06
2e+06
3e+06
Library size
MCF
4h
12h
24h
7d
BSF
c
PC2
60
40
20
0
−20
−10
0
10
20
30
PC1
expression of BARP genes
5
2.5
0
d
+2
0
-2
Genes
Early (MCF + 4 hpi)
Late (12 + 24 hpi)
e
ns
log2FC
p−value
p−value and log2FC
−log10P
40
30
20
10
0
−30
−20
−10
0
10
20
30
log2 fold change
total = 3043 variables
Tb927.8.7870
Tb927.11.1890
Tb927.3.3190
Tb927.11.7940
Tb927.4.1830
Tb927.3.1085
Tb927.3.2220
Tb927.8.4500
Tb927.3.4840
Tb927.1.700
Tb927.11.3410
Tb927.8.2450
Tb927.2.4590
Tb927.8.6650
Tb927.11.1910
Tb927.3.2510
f
Late (12 + 24 hpi)
Early (MCF + 4 hpi)
GO:0009190 cyclic nucleotide BP
GO:0009117 nucleotide MP
GO:0035556 intracellular signal transduction
GO:0007154 cell communication
GO:0055086 nucleobase-containing molecule MP
GO:0023052 signaling
GO:0019637 organophosphate MP
GO:0044281 small molecule MP
GO:0065007 biological regulation
GO:0051716 cellular response to stimulus
GO:0015877 biopterin transport
GO:0006139 nucleobase-containing compound MP
GO:0032042 mitochondrial DNA MP
GO:0046483 heterocycle MP
GO:0006094 gluconeogenesis
GO:0006793 phosphorus MP
GO:0044271 cellular aromatic compound MP
GO:0034641 cellular nitrogen compound MP
GO:0006461 protein complex assembly
GO:0005996 monosaccharide MP
GO:0006261 DNA-dependent DNA replication
GO:0005975 carbohydrate MP
GO:0034765 regulation of ion transmembrane transport
GO:0071163 DNA replication preinitiation complex assembly
GO:0032890 regulation of organic acid transport
GO:0016094 polyprenol BP
GO:0006438 valyl-tRNA aminoacylation
GO:0080144 amino acid homeostasis
GO:0006260 DNA replication
GO:0010608 posttranscriptional regulation of gene expression
GO:0051129 negative regulation of cellular component orga.
GO:0090150 establishment of protein localization to membrane
GO:0033036 macromolecule localization
GO:0051179 localization
GO:0007265 Ras protein signal transduction
GO:1900034 regulation of cellular response to heat
GO:0006567 threonine catabolic process
GO:0007155 cell adhesion
GO:0022610 biological adhesion
GO:0015833 peptide transport
GO:0048519 negative regulation of biological process
GO:0010948 negative regulation of cell cycle process
GO:0015791 polyol transport
GO:0008612 peptidyl-lysine modification to peptidyl-hypusine
GO:0065007 biological regulation
GO:0006890 retrograde vesicle-mediated transport, Golgi to ER
GO:0051641 cellular localization
GO:0034654 nucleobase-containing compound BP
4
3
2
1
0
-Log10 (p-value)

Supplementary Figure 6. Quality control of the single-parasite RNA sequencing experiment. (a, b) Violin and dot plots showing the number of genes detected across all libraries and library size of individual *T. brucei* parasites (metacyclic forms (MCF), n = 48; 4 h, n = 27; 12 h, n = 27; 24 h, n = 26; 7 d, n = 25; bloodstream forms (BSF), n = 31). The red lines represent the thresholds used for quality control. The BSF data set was prepared separately, indicated by the dashed line. Due to the overall high number of genes identified to be expressed in BSF cells, the upper threshold was increased to < 2100 genes. **(c)** Feature plot of the 142 transcriptomes that passed quality control (excluding BSF) showing the normalized expression of fly-stage specific BARP genes. BARP-genes are specifically expressed in epimastigote forms and three such parasites were excluded from the MCF transcriptomes. The color key from blue to red indicates low to high gene expression levels. **(d)** Heatmap showing the scaled expression levels of upregulated genes of early (MCF + 4 h) and late (12 hpi + 24 hpi) timepoints, as identified by SCDE (absolute log2 fold change > 2, z-score > 1.96). Each column represents a single parasite, each row represents an individual gene. The color key from purple to yellow indicates low to high gene expression levels. **(e)** Volcano plot illustrating differential gene expression between early (MCF + 4 hpi) and late (12 hpi + 24 hpi) time points. Genes upregulated in early time points are depicted on the right side of the graph, genes upregulated in late time points on the left. x- and y-axis show $\log_2$ fold change ($\log_2$ FC) against the adjusted -$\log_{10}$ p-value (-$\log_{10}$P) and dashed lines indicate the set thresholds. Genes with absolute $\log_2$ FC > 2 and p-value < 0.01 are considered as differentially expressed. **(f)** Biological process-associated gene ontology (GO) terms significantly enriched in early (MCF + 4 h) and late (12 hpi + 24 hpi) timepoints. GO terms were filtered with the Revigo webtool to avoid redundancies. Bars represent the level of significance of the term enrichment determined by Fisher´s exact test.

a

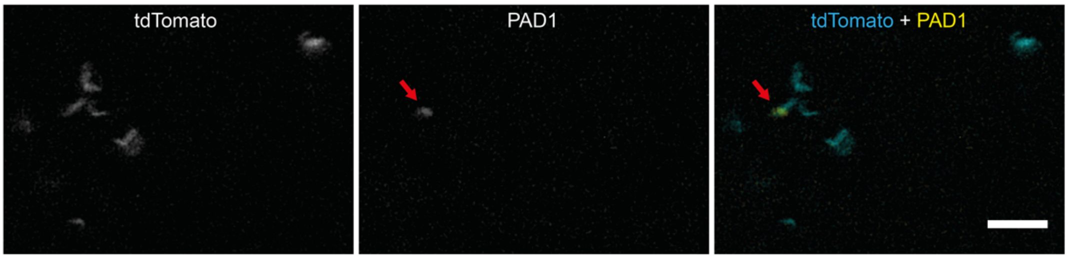

b

Days post infection	♂				♀			
	dissected	MG+	PV+	SG+	dissected	MG+	PV+	SG+
1	53	-	-	-	56	-	-	-
4	62	1	1	1	70	-	-	-
7	76	1	1	-	73	3	2	-

Supplementary Figure 7. Skin-residing parasites can develop into stumpy forms and are infectious to tsetse flies. (a) Stereo-fluorescence microscopy of tdTomato-expressing trypanosomes in a high-density skin equivalent (hdSE) infected for 7 days. The parasites also harbored a green fluorescent PAD1-marker fused to a nuclear localization signal as proxy for development to the stumpy stage. PAD1-positive parasites (GFP-positive nucleus, red arrow) were found in the hdSE at low incidence. Scale bar, 20 µm. **(b)** Infection of tsetse flies from hdSE-derived parasites. Trypanosomes were isolated from hdSEs at 1, 4, and 7 dpi, mixed with sheep blood, and fed to teneral tsetse flies. After 35 days, the flies were dissected and relevant organs were analyzed under a microscope and scored for the presence of trypanosomes. MG+, midgut positive, PV+, proventriculus positive, SG+, salivary glands positive.

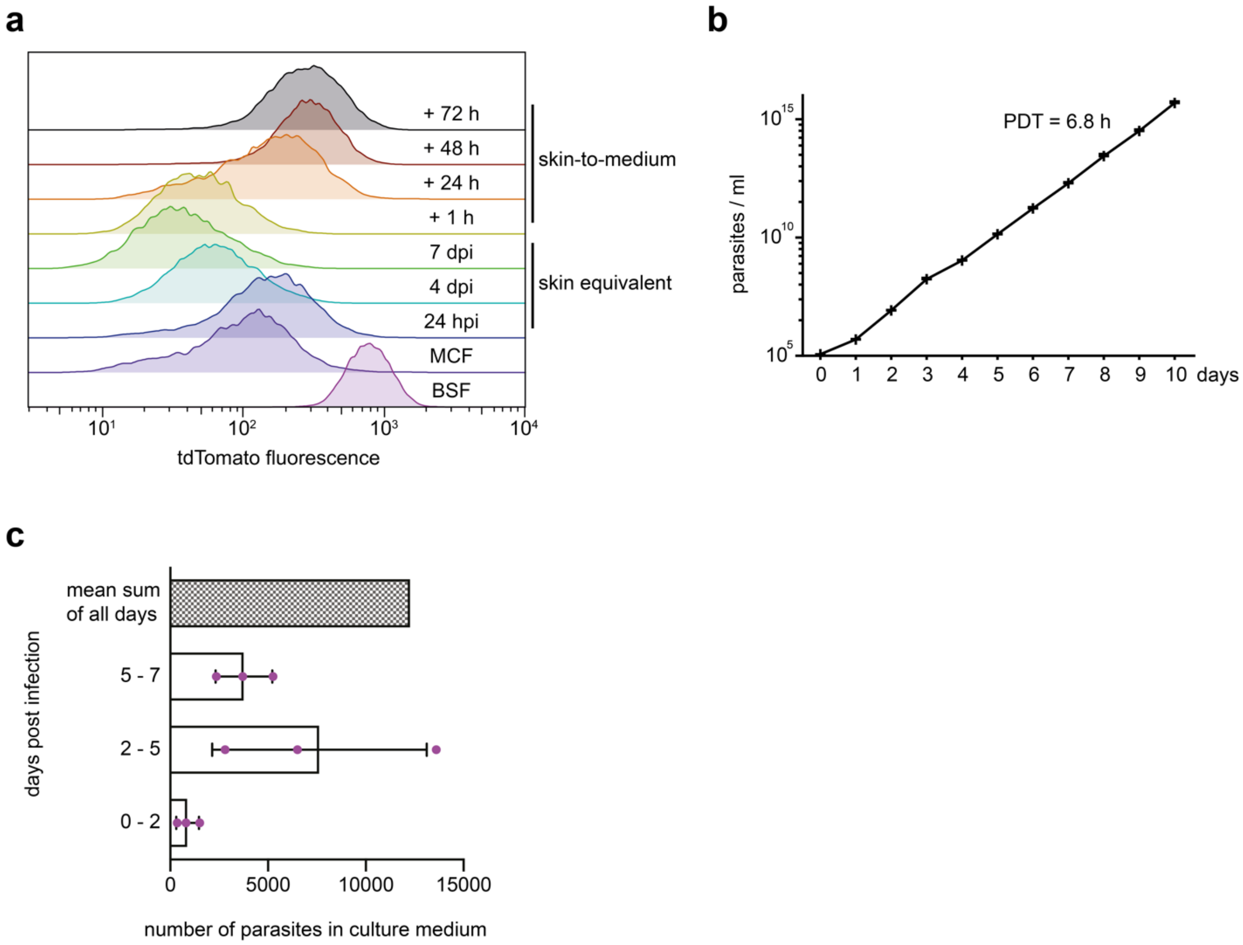

Supplementary Figure 8. Mean fluorescence intensity of tdTomato, growth of metacyclic trypanosomes under axenic conditions and parasites in culture medium of high-density skin equivalents. (a) Representation of flow cytometric histograms of the tdTomato fluorescence intensities of skin-residing trypanosomes compared to bloodstream forms (BSF), metacyclic forms (MCF), and skin-parasites at 7 dpi, which were transferred to culture medium for up to 3 days (skin-to-medium). **(b)** Growth and population doubling time (PDT) of MCF inoculated into HMI9 medium supplemented with methylcellulose. MCF were harvested from tsetse flies with a mature salivary gland infection by salivation. Data represent mean ± SD (n = 3 individual cultures). Source data are provided as a Source Data file. **(c)** Quantification of the parasites present in the culture medium during the culture of three infected hdSEs within one week. The culture medium was collected before the medium change on days 2, 5, and 7, centrifuged, and the number of trypanosomes was determined with a Neubauer chamber. Data of three replicates are shown. Source data are provided as a Source Data file.

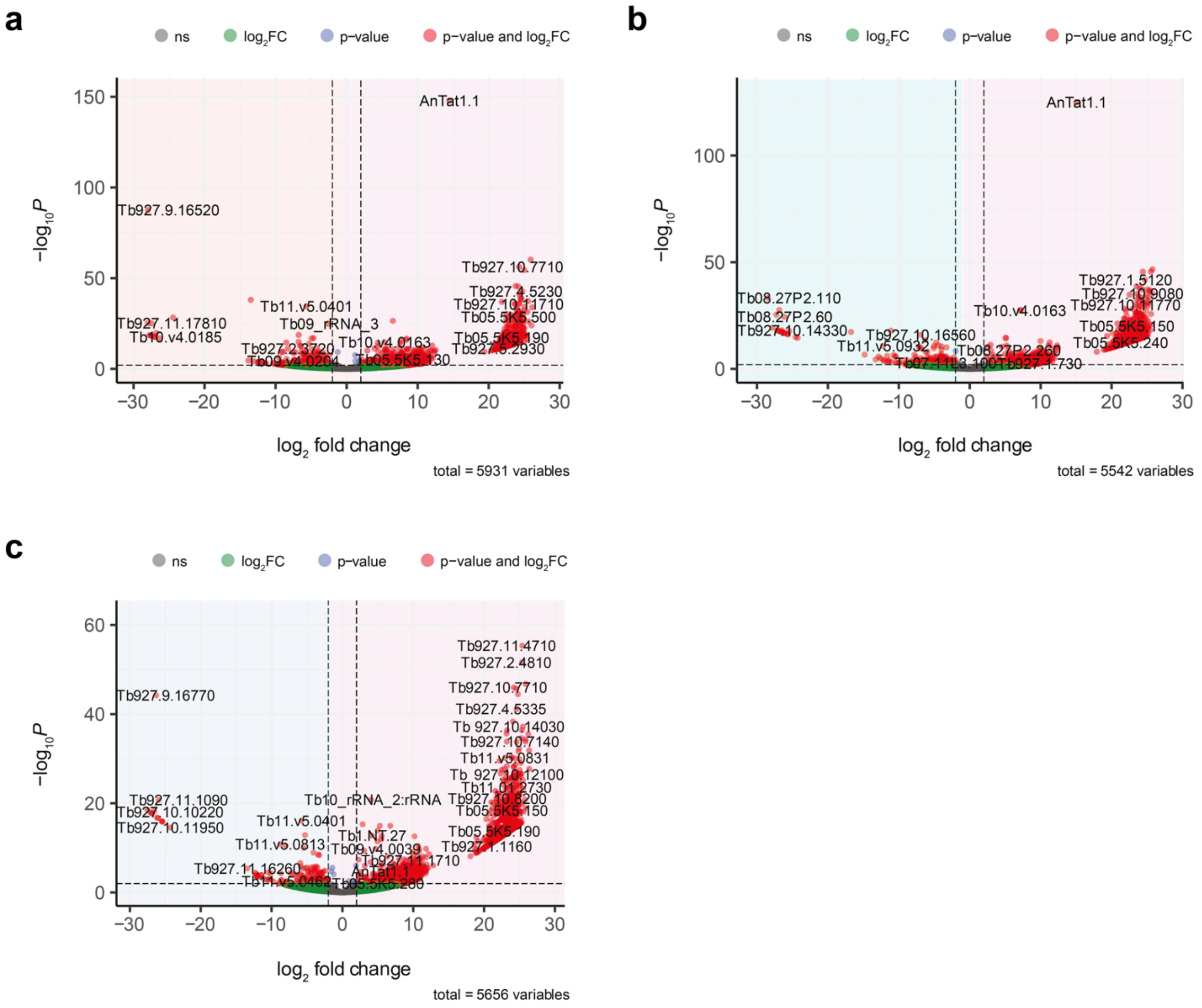

Supplementary Figure 9. Volcano plots illustrating differential gene expression in MCF, 24 hpi and 7 dpi each pairwise compared to BSF. Differential gene expression between **(a)** Bloodstream forms (BSF) vs. metacyclic forms (MCF), **(b)** BSF vs. 24 hpi, and **(c)** BSF vs. 7 dpi. Genes upregulated in BSF are shown on the right, genes upregulated in either MCF, 24 hpi or 7 dpi on the left. X- and y-axis represent $\log_2$ fold change against the adjusted $-\log_{10}$ p-value ($-\log_{10}$P), dashed lines indicate the set thresholds. Genes with absolute $\log_2$ fold change > 2 and p-value < 0.01 are considered as differentially expressed.

Supplementary References:

1 Feng, Z. *et al.* Investigation on the mechanical properties of contracted collagen gels as a scaffold for tissue engineering. *Artif Organs* **27**, 84-91, doi:10.1046/j.1525-1594.2003.07187.x (2003).

2 Reuter, C., Walles, H. & Groeber, F. Preparation of a Three-Dimensional Full Thickness Skin Equivalent. *Methods Mol Biol* **1612**, 191-198, doi:10.1007/978-1-4939-7021-6_14 (2017).

3 Macosko, E. Z. *et al.* Highly Parallel Genome-wide Expression Profiling of Individual Cells Using Nanoliter Droplets. *Cell* **161**, 1202-1214, doi:10.1016/j.cell.2015.05.002 (2015).

4 Philippeos, C. *et al.* Spatial and Single-Cell Transcriptional Profiling Identifies Functionally Distinct Human Dermal Fibroblast Subpopulations. *J Invest Dermatol* **138**, 811-825, doi:10.1016/j.jid.2018.01.016 (2018).

5 Tabib, T., Morse, C., Wang, T., Chen, W. & Lafyatis, R. SFRP2/DPP4 and FMO1/LSP1 Define Major Fibroblast Populations in Human Skin. *J Invest Dermatol* **138**, 802-810, doi:10.1016/j.jid.2017.09.045 (2018).

6 Wang, S. *et al.* Single cell transcriptomics of human epidermis identifies basal stem cell transition states. *Nat Commun* **11**, 4239, doi:10.1038/s41467-020-18075-7 (2020).

7 Meuter, S. & Moser, B. Constitutive expression of CXCL14 in healthy human and murine epithelial tissues. *Cytokine* **44**, 248-255, doi:10.1016/j.cyto.2008.08.009 (2008).
