# Supplementary material for: Vector-borne Trypanosoma brucei parasites develop in artificial human skin and persist as skin tissue forms
Source: Nat Commun. 2023 Nov 23;14:7660. doi: 10.1038/s41467-023-43437-2 (PMC10667367; doi:10.1038/s41467-023-43437-2)
Supplement: Supplementary file 3 — Description of Additional Supplementary Files [file 41467_2023_43437_MOESM3_ESM.docx]

**Description of additional supplementary files:**

**Supplementary Movie 1.** Related to Fig. 2. Natural transmission of *T.* *brucei* parasites to skin equivalents by tsetse fly. Three hdSEs were stacked on top of each other and subsequently tsetse flies with a mature salivary gland infection were allowed to bite into the stack.

**Supplementary Movie 2.** Related to Fig. 3. Tracking of trypanosomes in skin equivalents at various timepoints post infection. Skin equivalents were infected with *T.* *brucei* parasites by tsetse flies and cultured for 3 days. At the times indicated individual trypanosomes were recorded using a fluorescence stereomicroscope. Movies were acquired for 5 min at 4 fps and analyzed with Imaris software. Video plays at 20x speed.

**Supplementary Movie 3.** Related to Supplementary Fig. 7. Skin-resident trypanosome at 7 dpi expressing the stumpy marker PAD1. Skin equivalents were infected with *T.* *brucei* parasites by tsetse flies and cultured for 7 days. The movie was recorded with a fluorescence stereomicroscope for 1 min at 4 fps. Video plays at 3x speed.

# Supplementary Data 1. scRNAseq analysis of skin equivalents related to Fig. 1. Data was processed by CellRanger v3.1.0 (10x Genomics) and further analyzed using Seurat R package (version 3.1.4). The functions “NormalizeData”, “ScaleData”, FindVariableFeatures”, “FindNeighbors”, “FindClusters” were applied. “RunTSNE” was used for dimensionality reduction and genes with |‘avg_logFC’| > 0.25 and ‘p_val_adj’ < 0.05 determined by the Wilcoxon rank-sum test (“FindAllMarkers”) were considered as marker genes.

**Supplementary Data 2.** scRNAseq analysis of trypanosomes (MCF, 4 h, 12 h, 24 h, 7 d), related to Fig. 4. Differential gene expression analysis was conducted with DESeq2 (version 1.30.0) and SCDE (version 2.18.0; only used in early vs. late analysis). Features with an absolute log2 fold change > 2 and adjusted p-value < 0.01 (DEseq2) or a z-score > 1.96 (SCDE) were considered as differentially expressed.

**Supplementary Data 3.** Gene ontology (GO) term enrichment of trypanosomes (MCF, 4 h, 12 h, 24 h, 7 d), related to Fig. 4. The significance of the individual GO terms was determined by Fisher’s exact test using the GO enrichment tool on the TriTrypDB webserver. For early vs. late GO term analysis, genes with a z-score > 1.28 (SCDE) were used for enrichment.

**Supplementary Data 4.** scRNAseq analysis of trypanosomes (MCF/24 h vs. 7 d), related to Fig. 5. Differential gene expression analysis was conducted with DESeq2 (version 1.30.0). Features with an absolute log2 fold change > 2 and adjusted p-value < 0.01 (DEseq2) were considered as differentially expressed.

**Supplementary Data 5.** scRNAseq analysis of trypanosomes (MCF/24 h/7 d vs. BSF), related to Fig. 5. Differential gene expression analysis was conducted with DESeq2 (version 1.30.0). Features with an absolute log2 fold change > 2 and adjusted p-value < 0.01 (DEseq2) were considered as differentially expressed.

**Supplementary Data 6.** GO term enrichment of trypanosomes (MCF/24 h/7 d vs. BSF), related to Fig. 5. The significance of the individual GO terms was determined by Fisher’s exact test using the GO enrichment tool on the TriTrypDB webserver.

**Supplementary Data 7.** Top 20 VSGs expressed in trypanosomes (MCF, 4 h, 12 h, 24 h, 7 d), related to Fig. 6.
